# Deciphering the Interlinked CXCR4-Mediated Feedback Loop Among Signaling Pathways in Diabetic Wound Healing

**DOI:** 10.2174/0115733998335873241012161428

**Published:** 2024-11-29

**Authors:** Sandhanam Kuppusamy, Bedanta Bhattacharjee, Damanbhalang Rynjah, Pratap Kalita, Saikat Sen, Raja Chakraborty

**Affiliations:** 1 Department of Pharmacology, KMCH College of Pharmacy, Coimbatore, Tamil Nadu, 641048, India;; 2 School of Pharmaceutical Sciences, Girijananda Chowdhury University-Tezpur Campus, Assam, 784501, India;; 3 Department of Pharmaceutics, Pratiksha Institute of Pharmaceutical Sciences, Guwahati, Assam, 781026, India;; 4 Faculty of Pharmaceutical Science, Assam Down Town University, Sankar Madhab Path, Gandhi Nagar, Panikhaiti, Guwahati, Assam, 781026, India;; 5 Institute of Pharmacy, Assam Don Bosco University, Sonapur, Assam, 782402, India

**Keywords:** CXCR4, diabetic wound, angiogenesis, SDF-1, MAPKs, oxygen deprivation

## Abstract

Diabetic chronic wounds and amputations are very serious complications of diabetes mellitus (DM) that result from an integration factor, including oxygen deprivation, elevated reactive oxygen species (ROS), reduced angiogenesis, and microbial invasion. These causative factors lead to tenacious wounds in an inflammatory state, which eventually results in tissue aging and necrosis. Wound healing in DM potentially targets C-X-C chemokine receptor type 4 (CXCR4) regulates several signalling pathways. The CXCR4 signalling pathway integrated with phospholipase C (PLC)/protein kinase-C (PKC) Ca^2+^ pathways, stromal cell-derived factor-1 (SDF-1), and mitogen-activated protein kinases (MAPKs) pathway for enhancing cell chemotaxis, proliferation, and survival. The dysregulated CXCR4 pathway is connected with poor wound healing in DM patients. Therapeutic strategies targeting CXCR4-based molecules such as UCUF-728, UCUF-965, and AMD3100 have been shown to enhance diabetic wound healing by altering miRNA expression, promoting angiogenesis, and accelerating wound closure. This study indicates that CXCR4 participation in various signalling pathways makes it essential for understanding the healing of diabetic wounds. Using specific compounds to target CXCR4 offers a potentially effective treatment strategy to improve wound healing in diabetes. Our understanding of CXCR4 signalling and its regulation processes will enable us to develop more potent wound care solutions for diabetic chronic wounds. This report concludes that CXCR4's potential therapeutic targeting shows improvements in diabetic wound repair. This review will demonstrate that CXCR4 plays a major role in wound healing through its various signalling pathways. Targeting CXCR4 with certain agonist molecules shows a therapeutic approach to potentially increasing wound healing in diabetes. By enhancing our understanding of the CXCR4 signalling mechanism in future studies, we can develop more potential treatments for chronic diabetic wounds.

## 1 INTRODUCTION

Diabetes mellitus is a chronic endocrine condition that is becoming more common. It is defined as persistent high blood sugar levels triggered by deficiencies in insulin production, insulin mode of action, or both, which can have long-term health complications [1]. Delayed closure of diabetic wounds is believed to afflict around 25% of all diabetic individuals, frequently ending with lower limb amputation [1].

Diabetic wounds are caused by a combination of poor circulation, nerve damage, and susceptibility to infection [2]. The ‘diabetic wound’ term is also associated with a fusion of pathologies comprising hyperglycemia, diabetic neuropathy, Charcot’s neuro-arthropathy, peripheral vascular disease, osteomyelitis, foot ulceration, and limb amputation [3, 4]. Diabetic wounds in patients have continued to be a major health issue for the public and one of the main causes of hospitalization for diabetic patients [5]. Globally, the morbidity from diabetes is increasing at an uncontrollably high rate. Globally, more than 500 million people had diabetes in 2021. 783 million people worldwide are predicted to have diabetes by 2045. Diabetes is also expected to cause 6.7 million deaths in 2021. About 8.5% of people worldwide have diabetes-related morbidity. About 35 million people, or 12.6% of the total population, are diabetics in the United States alone. The epidemiology report of diabetic wounds in different continents is as follows: (i) in Africa, the prevalence is reported at 7.2%, (ii) Asia, the prevalence stands at 5.5%, (iii) Australia shows a prevalence of 2.0%, (iv) Europe's prevalence is reported at 5.1%, (v) in North America has the highest prevalence at 13%, and (vi) Oceania shows a prevalence of 3.0% diabetes wounds which is also depicted by a heat map that highlights the prevalence of diabetic wounds around the globe in Fig. (**1**) [6-9].

In the management of diabetic wounds, one of the major challenging phases is when it’s combined with ischemia. The generation of both resident and systemic cytokines in the presence of bacteria on the site of the wound might result in shock and systemic inflammatory response that necessitates appropriate management for the diabetic wound [10]. Based on the severity of the wound, various therapeutic approaches are applied. For instance, antimicrobial products and physical methods such as topical pharmaceuticals like chlorhexidine, 10% povidone-iodine solution, 5% acetic acid, and compounds having silver and hydrogen peroxide are commonly utilized. Furthermore, for minor and moderate infections, oral antimicrobial drugs that are mostly used are broad-spectrum which is combined with β-lactam or β-lactamase inhibitors such as sulbactam/ ampicillin, tazobactam/piperacillin, and clavulanic acid/ ticarcillin and the direct use of intravenous therapy for more severe infections [11]. Moreover, therapeutic management of diabetic wounds includes the removal of the bacterial biofilm and appropriate routine dressings with sucrose octa sulfate [12]. Other emerging treatments are the application of complement growth factors (belcaplermin-human platelet-derived growth factor), inflammatory regulators, blood and herbal products, hyperbaric oxygen therapy, skin replacement therapy, and stem cell therapy.

Because of the circumstances, the majority of the diabetic wound cases will heal which accounts for 60-80%, while 10-15% will remain operative and the other 5-24% of the cases will end up with amputation of the limb within a duration of 0.5 to 1.5 years of post-evaluation [13]. About 80% and 85% of non-traumatic amputations and wounds respectively account for diabetes [14]. Amputation is substantially higher with mortality which ranges from 13 to 40% at 1^st^ year to 39-80% at 5^th^ year. Moreover, about 50% of the individuals with amputation within 1.5 years will advance ulcerations and infections in the contralateral foot and an upsetting 58% will have contralateral amputation after the first amputation within 3-5 years [15].

Chronic wounds have a lot of common features, despite having diverse molecular etiologist, which include high concentrations of pro-inflammatory mediators, reactive oxygen species (ROS), senescent cells, and proteases which can often have a resolute infection and a lack of functional stem cells [16]. The microbes and platelet-derived factors (*i.e.* transforming growth factor-β (TGF-β)) or extracellular matrices (ECM) fragment molecules, trigger immune cell’s perpetual aggression due to frequent tissue damage and this ultimately intensifies and extends the pro-inflammatory cytokine cascade, which increases the concentration of proteases [17]. These proteases play different roles in different types of injuries. For instance, in acute wounds, the proteases are regulated by their inhibitory drug molecules, while in chronic wounds, the protease concentration surpasses the concentration of their particular inhibitors, which often leads to the deprivation of growth factors and their receptors and also it can also damage ECM [18]. The ECM destruction not only inhibits the wound from moving from the injury stage to the proliferative stage, but it also leads to the amplification of the inflammatory cycle by stimulation of more inflammatory cells into the wounded site and impedes the wound healing mechanism [19].

The migration of cells to the area of injury is regulated by chemokines, which are tiny (8-12 kDa) chemotactic cytokines. Several cells during injury such as macrophages, neutrophils, keratinocytes, fibroblasts, and endothelial cells release chemokines. Chemokines perform significant work in coordinating the inhibition or stimulation of angiogenesis at various stages of the healing process in wounds. Angiogenesis is stimulated during the period of inflammation to facilitate the passage of inflammatory cells into and out of the wound, but it is inhibited during the hemostasis step to allow for the development of clots which leads to chemokine imbalance and disrupts the wound healing approach, resulting in either extensive healing or increased scarring [19].

The C-X-C chemokine receptor 4 (CXCR4), a type of chemokine receptor is interrelated to tissue inflammation and wound healing. CXCR4 is a vital component in neurogenesis, vascular formation, cardio genesis, and germ cell growth along with abnormal phenomena including vascular development, and muscle remodelling [20]. CXCR4 is upregulated in the body during cellular injury, stress, hypoxic conditions, and vascular tissue damage and this dysregulation of CXCR4 interferes with the healing mechanism of wound [21].

CXCR4 treatment is a unique method that targets the CXCR4 receptor and has demonstrated encouraging results in helping diabetic people heal their wounds more quickly. This method promotes stem cell migration and multiplication, which is essential for tissue repair. CXCR4 therapy has several benefits over more techniques such as debridement, negative pressure wound therapy (NPWT), and hyperbaric oxygen therapy [22]. A study that compared CXCR4 therapy to NPWT, a widely used standard procedure. The study recruited diabetes patients with chronic, non-healing wounds. The results showed that, in comparison to patients receiving NPWT alone, patients treated with CXCR4 had much faster wound closure and lower infection rates. Enhancing stem cell recruitment and activation results in faster wound closure and decreases microbial contamination, following that it will greatly improve the quality of diabetic patients contains chronic wounds [22]. Following that CXCR4 novel therapy has evolved an increasing interest in stem cell migration and proliferation, which is the target of this technique. It is important to keep in mind that further research is need to completely understand the potential risks and long-term benefits of CXCR4 therapy [23]. In light of concerns about accessibility and cost-effectiveness, we also need to carefully evaluate its suitability for widespread clinical uses. The manuscript includes the review study algorithm flow chart in Fig. (**2**).

In this review, we will explore the importance of microbial invasion on wound healing, the importance, pathogenesis, regulation, implications of dysregulated CXCR4 in diabetic individuals, CXCR4 mediated signalling pathways, CXCR4 regulation, modulation, and treatment approaches and other standard therapies treatment for diabetic wound healing will be covered in this review.

## 2 CONTRIBUTION OF MICROBIAL INVASION IN DIABETIC WOUND HEALING

Numerous findings demonstrate that microbes have the potential to penetrate wounds, certain data can substantiate the encouraging benefits of microbial invasion in wound recovery. In 2021, Wang *et al*. reported that an adequate quantity of *Staphylococcus aureus*, when administered to wounds at precise periods, initiated hair shaft rejuvenation Furthermore, severe wounds in particular pathogen-free (PPF) mice healed more easily compared to germ-free (GF) mice, prompting enormous attention on the link underlying microbial invasion and wounds [24].

A comparable study executed in 2021 observed that applying *Staphylococcus epidermidis* accelerated wound recovery whereas antibiotic therapy impeded it [25]. An investigation from 1983 demonstrated that purposeful contamination of *Staphylococcus aureus* in mild wounds in rodents improved collagen production and linkage in wounds, facilitating wound recovery, which is a substantial correlation with several microbes injected [26].

This ostensibly conflicting data suggests that the role of microbes in wounds cannot be easily extrapolated and demands extensive research [27, 28]. Although rodents are frequently used as subjects to investigate the course of wound healing, there are currently some challenges expressed concerning the model's accuracy in forecasting the healing of wounds in humans [29]. Rats and mice exhibit thinner dermal and epidermal layers compared with humans, and as a result, their wounds heal predominantly by contraction instead of re-epithelialization [30]. Pigs are additionally employed since they are regarded as the finest approximation for human skin concerning of anatomical and healing characteristics [31].

Anti-microbial peptides (AMPs) produced by epidermal adherent microbes might have a significant role in the recuperation of wounds [32]. Even though *Staphylococcus aureus* is a common microbe that lives on the skin's surface, linked to a higher chance of skin infections. Large amounts of *Staphylococcus aureus*, on the other hand, are thought to slow down wound healing. Previous studies have shown that giving small amounts of *Staphylococcus aureus* can promote skin rejuvenation and expedite wound closure. By killing off similar harmful bacteria, *Staphylococcus aureus* can also speed up wound healing. Research shows that when *Staphylococcus aureus* settles on a cut, *Streptococcus anginosus* is not present in large amounts. This prevents complex cytokine storms from developing in the wound quickly, which lowers neutrophil depletion factors and interleukin-17 (IL-17) and speeds up the healing process [33].

Because *Pseudomonas* affects different signaling pathways in the host organism, it seems likely that the microbiome will not have the same effect on the host. Small amounts of *Pseudomonas aeruginosa* get into the epithelial tissue and cause keratinocytes to multiply. They also make cytokines, such as vascular endothelial growth factor (VEGF), which helps the process of angiogenesis happen inside wounds. Since epidermal microbes can have different effects on signaling pathways, it seems reasonable to think that the right amount of certain microbe species could help keep the epidermal microenvironment stable [34].

Investigators revealed that when mice experience trauma, nucleotide-binding oligomerization domain-2 (NOD2) is increased, improving wound recovery. NOD2 operates as a motif detection receptor, predominantly recognizing antimicrobial peptides and stimulating the host immune mechanism [35]. In conclusion, an adequate amount of microbial invasion may facilitate wound recovery by sustaining moderate inflammatory conditions; nonetheless, excessive microbial colonization seems to hinder wound healing [34]. It is interesting to contemplate strategies to manage the fragile interaction involving microbiota and the epidermis as well as how to maximize the positive aspects of microbes while minimizing the adverse consequences.

## 3 EXPLORATION OF CXCR4 IN DIABETIC WOUND HEALING

### 3.1 Regulation of CXCR4 Expression

CXCR4 was first discovered in peripheral blood leukocytes. CXCR4 is a 7-transmembrane G-protein coupled receptor that is extensively distributed in a variety of cell types, such as lymphocytes, cancer cells, endothelium, epithelial, and hematopoietic stem cells [36]. In addition to its established roles in immunological responses and haematopoiesis, CXCR4 is necessary for serving physiologic functions. For instance, studies have shown its involvement in neurogenesis, vascular development, cardio genesis, and germ cell growth [37]. Two transcription factors primarily control the basal expression of CXCR4: (i) the critical regulator Ying Yang-1 (YY1) and (ii) the nuclear respiratory factor-1 (NRF-1), which stimulates receptor transcription [38]. The basic helix-loop-helix (bHLH) binding sites, SRY-Box transcription factor-17 (SOX17) binding sites, Pit-Oct-Unc (POU) binding sites, Forkhead Box (FOX) binding sites, and palindromic Smad binding sites are among the important transcription factors whose binding sites are present in the proximal enhancer region of CXCR4 and contribute to its upregulation. These proximal enhancer area binding sites are substantially conserved amongst the orthologs of CXCR4 in humans, chimpanzees, mice, and rats, according to comparative genomic investigations, indicating their importance for basal transcriptional control [39]. Through binding to various Gα subunit families, CXCR4 can signal. Adenylate cyclase (AC) is stimulated by CXCR4 connected to Gαs, whereas AC is inhibited by CXCR4 bound to Gαi. AC catalyses the transformation of adenosine triphosphate (ATP) into cyclic adenosine monophosphate (cAMP), which controls MAPK signalling and activates cAMP-dependent protein kinase [40]. Calcium mobilization was shown to be mostly adversely regulated by G-protein-coupled receptor kinase 2 (GRK2), GRK6, and arrestin-3, while GRK3, GRK5, and arrestin-2 had no effect, according to an analysis of CXCR4 signalling in human embryonic kidney 293 (HEK293) cells [41]. Different signalling chemicals, like calcium, cytokines (IL-2/4/7/10/15 and TGF-β), cyclic-AMP, and growth factors (VEGF, and bFGF), all cause an upregulates in CXCR4 transcription [42]. Conversely, the expression of CXCR4 has been seen to be downregulated in the presence of several other cytokines, including tumour necrosis factor-alpha (TNF-α), interleukin-1β (IL-1β), and interferon-1γ (INF-γ) [43]. Certain post-translational modifications (PTMs) may also give the receptor unusual characteristics. CXCR4 can modify receptor intracellular distribution, signalling, and activities once it is produced by phosphorylation, glycosylation, ubiquitination, and sulfation. Glycosylation or tyrosine sulfation at Asn11 on CXCR4 appears to be essential for CXCL12 interaction with the receptor in mammalian cells. Furthermore, CXCR4 is glycosylated at regions Asn11 (g1) and Asn176 (g2) inside the N-terminal domain and the secondary interstitial loop, respectively [44]. One of the most significant CXCR4-PTM is represented by phosphorylation. The process by which CXCR4 is rapidly phosphorylated on its C-terminal motif and internalized subsequent interaction with CXCL12/SDF-1 is known as desensitization. After internalization, the E3 ubiquitin ligase AIP4 can either recycle CXCR4 back to the cell surface or cause lysosomes to break it down. CXCR4 location extends beyond the cell surface, as numerous cell types exhibit substantial intracellular receptor storage [45].

### 3.2 CXCR4 Signalling Pathways

CXCR4 includes several physiological and pathological processes. There are two steps involved in the binding process between CXCL12/SDF-1α and CXCR4 [46]. Stronger chemokine binding to a receptor site is caused by a conformational shift in the receptor caused by the first CXCR4-CXCL12 interaction at the non-cellular region. Subsequently, a second structural shift occurs in the receptor, causing the Gα subunit to separate from the Gβ/Gγ dimer and activate the intracellular trimeric G protein [47]. Pertussis toxin (PTX) impairs GPCR/Gi coupling by adenosine diphosphate (ADP)-ribosylation of the Gαi component, impeding CXCR4-mediated activities [48]. Stromal cell derived-factor-1 (SDF-1) mediated signalling pathways *via* CXCR4 are elucidated in Fig. (**3**).

Gαi, once activated, it phosphorylates the adaptor protein Shc to modulate cell cycle progression by inhibiting adenyl cyclases and resulting in the generation of cAMP [49]. It also promotes the functioning of Src family tyrosine kinases, which trigger the Ras/Raf/MEK/ERK cascade [50]. Gβγ and Gα subunits can both activate phosphoinositide-3 kinase (PI3Ks), which in turn mediate CXCR4-oriented migration. PI3Ks phosphorylate numerous focal adhesion markers (talin, FAK, zyxin, and p130Cas, *etc*) and Akt to control cell motility, gene transcription, and cell adhesion [51]. Gβ/Gγ complexes initiate phospholipase-C (PLC) activation, which hydrolyses phosphatidylinositol 4,5-bisphosphate (PIP2) to produce inositol 1,4,5-trisphosphate (IP3) and diacylglycerol (DAG) [52]. The generation of IP3 causes the intracellular stores of Ca^2+^ to be mobilized, while DAG stimulates the activation of protein kinase-C (PKC) and mitogen-associated protein kinase (MAPK) [53]. CXCR4 can also transmit through other Gα proteins, including Gαs, Gαo, and Gαq in addition to its well-established connection to Gαi. Furthermore, CXCR4 has been shown to initiate a signal pathway that is not dependent on G proteins [40]. CXCR4 activates G-protein coupled receptor kinases (GRKs) after interacting with CXCL12, resulting in site-specific phosphorylation at the C-terminus and associates with β-arrestins. The uncoupling of CXCR4 from G protein and the subsequent induction of receptor internalization are caused by arrestin recruitment [54]. By raising GRK2 activity, c-Src, PKA, and PKC can encourage GPCR internalization. PKC directly phosphorylates the C-tail of CXCR4 at various serine sites, facilitating receptor internalization. However, β-arrestins attachment to CXCR4 also acts as a platform for several downstream modulators [55]. CXCR4-based p38 and extracellular signal-regulated kinase (ERK) stimulation are enhanced by β-arrestin-1/2, which in turn accelerates cell motility [56]. After CXCL12 interaction, Janus kinases (JAK2 and JAK3) quickly attach to and activate CXCR4 causing it to oligomerize and become phosphorylated. The G-protein-independent Janus kinase/Signal transducer and activator of transcription (JAK/STAT) intracellular signalling pathway is activated by CXCR4 oligomerization. When active, STATs control several biological functions, such as the mobilization of Ca^2+^ from intracellular reserves and the transcription of several target genes following its nuclear translocation [57]. Like other chemokine receptors, CXCR4's best signaling is exerted when integrated within membrane rafts. CXCR4 is facilitated by various modulators, including adhesion molecules, fibrinogen, fibronectin, and cytokines [57].

### 3.3 Implication of CXCR4 in Diabetic Wound Healing

The only intrinsic mediators of CXCR4 may be a cluster of differentiation 4 (CD4), SDF-1, and a cluster of differentiation 74 (CD74). Both biological activities including the development of germ cells, cardiogenesis, neurogenesis, and angiogenesis, as well as degenerative activities including muscle remodeling are significantly upregulated by CXCR4 [58]. Stress, injury, and vascular tissue damage can all cause an increase in CXCR4 [21]. The release of SDF-1 can rise dramatically in response to damage to tissues including the heart, liver, brain, and kidney. As a result of trauma, bone marrow-derived CXCR4-positive stem cells are stimulated to produce initial tissue biomarkers and take part in wound healing and regeneration [59].

The SDF-1/CXCR4 system controls the main physiological processes that help wounds heal, such as the movement of inflammatory cells to the site of the injury, the growth of new cells to heal the wound, and the build-up of collagen to change the structure of the wound [60]. The SDF-1/CXCR4 signalling pathway activates the other signalling pathways, including NF-κB, which controls intracellular transcription, Ca^2+^ efflux, and cell survival; and the PI3K/Akt, JAK/STAT, and mammalian target of rapamycin (mTOR) pathways [61]. The basic processes of cell survival, proliferation, chemotaxis, apoptosis, and differentiation can all be regulated by the SDF-1/CXCR4 signal axis, which can also promote angiogenesis in specific conditions. It thus has a significant impact on the healing of wounds [62]. Yang *et al.* investigated that overexpressing CXCR4 in bone marrow-derived mesenchymal stem cells (BMSCs) speeds up cutaneous wound healing in a mouse model. The BALB/c mice took 14 days to cure their wounds, but the severe combined immunodeficiency (SCID) and nude mice took 19.83 days and 17.16 days, respectively. Around the wounds of female BALB/c mice exposed to ^60^Co radiation, male BMSCs were found, and these cells exhibited CXCR4 overexpression, which sped up the healing process [63]. Guo *et al*. investigated that in diabetic wounds, the range of SDF-1 surrounding the wound edge greatly increased, and increased the healing of the wound by upregulation of wound closure time [64]. Mesenchymal stem cells (MSCs) have been investigated by Wei *et al*. to be able to overexpress CXCR4 (CXCR4-MSC), which facilitates better intervertebral disc (IVD) regeneration and increased motility of the cells. The expression of CXCR4 on natural killer cells can be regulated by activated tissue-resident MSCs to facilitate vascular regeneration [65]. The research information presented above indicates that CXCR4 and its activities show the upregulation of angiogenesis, wound closure, and diabetic wound tissue regeneration.

### 3.4 Dysregulation Signalling Pathways in Cases of Recurrence of Diabetic Foot Ulcers

For the small number of individuals whose ulcers cannot heal or for whom healing would be a redundant medical or social burden, a curative strategy that decreases the complexity of treatment and lowers the risk of sepsis and hospitalization may be desirable. The high ulcer recurrence rates are caused by either physical, biological or by both of the factors. After healing, many of the triggering conditions that initially caused ulcers such as foot peripheral vascular disease, peripheral neuropathy, deformity, and increased plantar stress usually remain unresolved [66]. Surgical procedures can enhance the blood flow to the foot and foot structure. Still, it does not address the significant concurrent neuropathy that is the permissive factor in the injury that is instigated by recurrent stress and results in ulceration and inflammatory conditions. Consequently, an ulcer might still be caused by a combination of these physical conditions [67].

However, evidence of cellular senescence, an undetectable underlying force has long been accumulated in the pathophysiology of wound chronic conditions, and ulcer recurrence. As a result, aging manifests itself in several interconnected ways at the cellular, molecular, and organismal levels [68]. Senescence is a mechanism in the cells that causes proliferative detainment and is characterized by changes in morphology, metabolic reprogramming, the application of a complex pro-inflammatory transmissible secretome, epigenetic remodelling, susceptibility to apoptosis, and elevated autophagy. Numerous variables that contribute to cellular senescence are overexposed in the diabetic location, which eventually results in organismal aging with recognized nosogenic implications [69].

An interconnected and perpetual cycle is produced by the overproduction of ROS and the widespread oxidative damage on multicellular targets, particularly mitochondrial components. Many effector mechanisms cause cell senescence and proliferative stoppage when mitochondrial and genomic DNA damage, involving telomeric areas, develops [70]. The beginning of the cellular senescence phenotype caused by the effects of a high glucose contents has been linked to two main fundamental operators both p53-p21 and pRb-p16INK4a-p21Waf1 [71]. Furthermore, p53 is associated in the control of cell cycle disruption and senescence, according to scientific reports. Recent research has demonstrated that telomeric damage initiates a p53-dependent pathway that suppresses the expression of the gene encoding peroxisome proliferator-activated receptor gamma co-activator-1α (PGC-1α), ultimately leading to mitochondrial damage [72]. PGC-1α is an expert modulator of mitochondrial biogenesis and operations, that encompassing ROS detoxification and oxidative phosphorylation. The orchestration of the DNA damage response (DDR) impairs other cognate proteins implicated in mitochondrial biosynthesis and function, hence worsening the degradation of the mitochondrial system [73].

Similarly, pRb (retinoblastoma protein) is essential for the initial phase of senescence. p16INK4a and p21Waf1 work together as cell cycle inhibitors to maintain pRB in its hypo-phosphorylated, growth-inhibitory state of motion. Particularly, fibroblasts produced from non-healing DFU showed nuclear upregulation of p53 phosphorylated on serine-15 and p21Cip1. This observation offers evidence to the assertion that granulation tissue fibroblasts proliferative hindrance promotes fibrovascular stagnancy and cellular senescence [74].

It can be noted that even after closure of the DFU with the management with mesenchymal cells (MSCs) therapy in the initial stage, chances of recurrence may occur. After 3 years of observance on the closure of the initial wound, full reinstatement of typical anatomical factors of the skin is not achieved. The partial closure of the wound may lead to recurrence of DFU. MSCs alone may not be able to completely heal the DFU area, but it can be employed as an adjuvant with other innovative strategies like patient education, prophylactic measures (foot care, therapeutic footwear), and patient diet to achieve full skin healing for long term treatment [75]. All of these behavioural characteristics contribute to an increased risk of diabetic foot ulcer recurrence.

## 4 CXCR4-MEDIATED INTRACELLULAR SIGNALLING

### 4.1 Activation of SDF-1/CXCR4 Signalling Cascade

In stromal cells of the bone marrow, SDF-1 is extensively expressed. To heal wounds, SDF-1 attracts stem cells and controls their differentiation. In the injured tissues, TGF-β and VEGF are two examples of the growth factors and cytokines that regulate repair [76]. SDF-1 is a chemokine peptide that is rapidly inactivated by matrix metalloproteinase-2 (MMP-2), an enzyme that is very prevalent in inflammatory environments [77]. The chemokine SDF-1 is involved in both angiogenesis and wound healing. SDF-1 binds to the G-protein-coupled CXCR4 and CXCR7 to carry out its biological activity. Stem and progenitor cells, such as hematopoietic stem cells, MSCs, and other cells expressing CXCR4, are recruited and mobilized by SDF-1 [78]. A growing body of research indicates that SDF-1 interaction with CXCR4 and CXCR7 is essential for preserving MSC survival and proliferation in the face of stress. Research has demonstrated that SDF-1 can increase proliferation, MSC migration, secretion, and anti-apoptotic ability while also enhancing cell transplant survival [79]. The skin wound-healing process is a dynamic, intricate process that is meticulously planned, with sequence events that overlap between phases. Numerous cell types and other variables are involved. CXCR4 is among the most significant [80]. Regeneration cannot occur without inflammation since tissue damage frequently results in acute inflammation and tissue regeneration, and the three are inextricably linked. To preserve tissue homeostasis, inflammation primarily serves to eradicate pathogen infiltration and eliminate necrosis in the tissue [81]. Numerous investigations have demonstrated that inflammatory cells express CXCR4, which enhances their capacity to migrate to ischemic regions and aids in tissue healing and revascularization [82]. Simultaneously, certain lymphoid or non-lymphoid organs constitutively express SDF-1, the ligand of CXCR4, which contributes to inflammation [45]. It appears that neutrophils play a crucial role in providing CXCR4/SDF-1, which is required for wound healing. The first cells to be recruited during the inflammatory response are neutrophils [83]. CXCR4 neutrophil subsets, which quickly relocate to the site of inflammation, serve as the first line of defence against invasive pathogens during the acute inflammatory response. Monocytes then reinforce the inflammatory response. To return to the bone marrow and eventually be ingested and destroyed by the local macrophages, aged neutrophils can upregulate the expression of CXCR4 on their surface. This helps to reduce inflammation around wounds [84]. Tissue damage in wild-type mouse models, SDF-1 is increased in the wound epidermis and attracts leukocytes that express CXCR4 to the site of injury. Leukocytes then move into lesions to eliminate debris and kill invasive microbes [85]. Immune cell recruitment is necessary for the early wound healing response to begin, but certain types of immunity suppression can hasten the regeneration process that follows. During the inflammatory process, immune cells congregate at the site of the wound and create growth factors, including fibroblast growth factor (FGF), which promotes fibroblast rebuilding, re-epithelialization, and extracellular matrix remodeling in addition to fighting off invasive germs [86]. Inflammation can be induced *via* the SDF-1/CXCR4 axis, leading to fibrosis and the production of scars [86]. By using the CXCR4 inhibitor AMD3100, mice skin wounds and appendage regeneration can be repaired without scarring and the following recruitment of leukocytes expressing CXCR4 can be greatly reduced. Additionally, AMD3100 has been shown to enhance scar formation and wound healing in diabetic mice [87].

### 4.2 Role of PI3K/Akt, MAPKs, PLC/PKC-Ca^2+^, Wnt/β-catenin, and TGF-β Pathways

#### 4.2.1 PI3K/Akt Pathway

A lot of people are interested in this signalling pathway because it plays an important role in controlling many biological processes, such as glucose metabolism, apoptosis, and angiogenesis. When this pathway is activated, Akt is phosphorylated and turned on. Akt then affects many downstream targets that are involved in different cellular processes. PI3K/Akt pathways are out of whack in diabetic wounds because of high oxidative stress and poor insulin signalling [88, 89]. In diabetes, the PI3K/Akt pathway is often slowed down. This is mostly because of increased oxidative stress and poor insulin communication. One thing that sets type 2 diabetes apart is insulin resistance, which makes the route less active [90]. This interference stops Akt from becoming phosphorylated and turning on, which hurts communication further down the line. Also, chronic hyperglycemia in diabetes causes oxidative stress, which in turn creates ROS. These have the direct power to stop Akt from working [91]. Changes in the PI3K/Akt pathway caused by diabetes make it harder for cells to divide, stay alive, and make new blood vessels. This makes it take longer for wounds to heal [92]. The PI3K/Akt pathway manages cell movement by moving keratinocytes to help re-epithelialize and increase the number of fibroblasts and their ability to make extracellular matrix [93]. Extracellular ligands that bind to receptor tyrosine kinases (RTKs) bring in and activate PI3K. This then phosphorylates PIP2 to make PIP3 [94]. Some parts of the PI3K/Akt signaling system, like mTOR and glycogen synthase kinase-3 (GSK-3), affect how people with DM use glucose and how quickly wounds heal (Fig. **4**) [95].

The PI3K/Akt pathway has a significant impact on the healing of wounds. Through its control of cytoskeletal dynamics and focal adhesion turnover, the route facilitates efficient cell migration into the wound bed, hence promoting re-epithelialization and tissue repair [96]. The development of lamellipodia and filopodia is stimulated by Akt activation, which aids in cell movement and wound closure [96]. In addition, the route ensures that there are enough cells for tissue repair by preventing apoptosis and encouraging cell cycle advancement. Furthermore, the PI3K/Akt pathway controls cell differentiation, encouraging progenitor cells to differentiate into specialized cell types that are needed for tissue repair, such as fibroblasts, endothelial cells, and keratinocytes [97].

#### 4.2.2 MAPK Signalling Pathway

The mitogen-activated protein kinase (MAPK) signalling pathway is yet another important and highly conserved intracellular signalling cascade involved in wound healing [98]. Three primary subfamilies of MAPK genes have been identified, which are involved in signal transmission and cellular response regulation: (i) p38-MAPKs, (ii) jun N-terminal kinases (JNKs), and (iii) extracellular signal-regulated kinases (ERKs) [98]. The MAPK pathway is essential for wound healing because it controls inflammation, proliferation, cell survival, immune response, and differentiation during tissue repair. The deregulation of the MAPK pathway is caused by two factors: persistent inflammation and oxidative damage brought on by hyperglycaemia [99]. Remodelling, collagen synthesis, immune cell activation, Inflammatory reactions, and re-epithelialization are all adversely affected by this imbalance. Understanding how the MAPK pathway affects these cellular processes is very important because it can help find new treatment targets that could improve wound healing rates and return the route to normal which is shown in Fig. (**4**) [100]. Aberrations in MAPK signalling have been reported in diabetic foot ulcers, which may contribute to compromised wound healing. Diabetes-related persistent hyperglycaemia, which inhibits keratinocyte migration, is one of the main factors causing diabetic wound healing delays [101]. It has been suggested that during wound healing, the p38/MAPK pathway regulates autophagy and influences keratinocyte migration. High-glucose environments, the p38/MAPK pathway was down-regulated together with autophagy inactivation, which prevented keratinocyte migration [101]. Furthermore, it has been noted that pro-inflammatory cytokines like TNF-α and IL-1β cause increased activation of the p38 MAPK and JNK pathways, which can result in chronic inflammation and impede the repair of wounds [102]. The significance of the MAPK pathway in diabetic wound healing has been shown by the demonstration of how negative-pressure wound therapy can reduce inflammation and accelerate wound healing in individuals with diabetic foot problems by down-regulating the MAPK/JNK signalling pathway. Prolonged and chronic inflammation is considered a major factor impeding the healing process of diabetic wounds [103]. Exosomes are new players in the intercellular communication process and are essential for controlling the inflammatory immunological microenvironments around diabetic wounds [104]. A recent investigation found that mesenchymal stem cell-derived exosomes, or MSC-expos, can shield β cells from hypoxia-induced death. The presence of miRNA-21, which reduces endoplasmic reticulum (ER) stress and inhibits p38-MAPK activation, is thought to be responsible for this protective effect [105]. MSC-exosomes have a lot of potential for aiding in the healing of diabetic wounds. Significant effects of the MAPK pathway are seen in several cellular processes necessary for wound healing [106]. The ERK pathway is activated in a way that facilitates keratinocyte migration, proliferation, the creation of granulation tissue, and wound closure. The JNK pathway controls the inflammatory response and immune cell recruitment by regulating the synthesis of pro-inflammatory cytokines like TNF-α and IL-1β, chemokines, and MMP [107]. MAPK effectors, such as MAPK kinases 6 (MKK6) and its direct downstream target kinase, p38, mediate a non-canonical pathway that regulates fibroblast activation, myofibroblast differentiation, and collagen synthesis, all of which are essential for the contraction of wounds and reconstruction [108].

#### 4.2.3 Diacylglycerol-protein Kinase C (DAG-PKC) Activation

Several mechanisms, including the stimulation of the diacylglycerol-protein kinase-C (DAG-PKC) pathway, non-enzymatic glycation, polyol pathway flow, and oxidative stress, have been postulated to explain the deleterious effects of high glucose levels [109]. Cell proliferation, endothelial permeability, leucocyte adhesion, cytokine activation ECM synthesis/turnover, angiogenesis, vasoconstriction, and are all impacted by the DAG-PKC pathway, which is essential to the functioning of the vascular system which is depicted in Fig. (**4**) [109]. Vascular tissues during diabetes contain higher amounts of total DAG. There are several ways that this increase in DAG levels might happen, including phosphatidylinositide hydrolysis, PLC-mediated phosphatidylcholine metabolism, or *de-novo* production from glycolytic intermediates such as glycerol-3-phosphate and dihydroxyacetone phosphate [110]. Increased glucose incorporation into the glycerol backbone of DAG has been shown by metabolic labeling studies [111]. Glycerol-3-phosphate acyltransferase and monoacylglycerol-3-phosphate acyltransferase catalyze sequential acylation in the DAG production. DAG, a substance that includes both unsaturated and saturated fatty acids, enhances PKC activation [112]. Among them, 1-stearoyl-2-arachidonyl-sn-glycerol may be the most active, but 1-palmitoyl-2-oleoyl-sn-glycerol is a substantial amount. Furthermore, elevations in antioxidants like H_2_O_2_ brought on by hyperglycaemia may stimulate the DAG-PKC pathway by either solely activating PKC or enhancing DAG synthesis [112]. The majority of higher DAG levels under hyperglycaemic settings are made up of palmitate fatty acids, which lends credence to the idea that phospholipase-D (PLD) pathways or new synthesis are the primary causes of raised DAG levels rather than PLC's rapid production [113]. Numerous difficulties related to diabetes have been linked to enhanced PKC activation. These issues include fluctuations in enzymatic activity, such as MAP kinase, Na^+^-K^+^-ATPase, and cytosolic phospholipase A2 (cPLA2), as well as changes in blood flow, angiogenesis, basement membrane thickening, cell proliferation, vascular permeability, and ECM expansion [114].

#### 4.2.4 Wnt/β-catenin

The Wnt pathway is one of the developmental conserved pathways with an important role in stem cell maintenance, cell polarity, embryonic development, differentiation, and lineage specification. In the human genome, nineteen Wnt genes have been recognized and stipulated to initiate either the Wnt/β-catenin, Wnt/Ca^2+^ signalling pathway or Wnt/planar cell polarity (Wnt/PCP) [115]. Furthermore, the Wnt/β-catenin signalling pathway is stimulated by the binding of Wnt ligands to frizzled transmembrane receptors. Upon the process of repairing the wound, hypoxia activates the Wnt/β-catenin pathway which leads to the increase in Wnt ligands' production and through upregulating of the gene expression associated with cellular migration, proliferation, stem cell activation, and angiogenesis improves the wound healing process. However, when the overexpression of the pigment epithelium-derived factor (PEDF) impaired the Wnt/β-catenin pathway in diabetic mouse models, it hindered the angiogenesis, in repairing of wounds. Vascular endothelial cells direct themselves in reaction to prolonged high glucose levels through the action of effectors of the Wnt/β- catenin pathway. One of the reasons of diabetic ulcer's unpredictable behaviour is the downregulation of the Wnt/β- catenin pathway, which might be brought on by diabetes mellitus-induced reductions in R-spondin 3 (Rspo-3) proteins [116]. So, various techniques to improve wound healing, including naturally derived pharmaceuticals, tetrahedral framework nucleic acids, stem-cell-based therapies, and shock wave therapy, have been shown to stimulate the Wnt/β-catenin pathway [117].

#### 4.2.5 TGF-β

The transforming growth factor-β (TGF-β) family includes TGF-β1-3, activins, and bone morphogenic proteins. TGF-β signalling begins when the TGF-β binds to TGF-β receptor-II (TβR-II). Afterward, TβR-II assembles into a heterotetrametric complex with the two pairs of TβR-II and one pair of TGF-β receptor-I (TβRI). The TβRI is subsequently triggered by phosphorylation of serine/threonine residues in the glycine/serine area [115]. Subsequently, the phosphorylation of the suppressor of mothers for decapentaplegic Smad2 and Smad3 is triggered by the activated TβRI. Finally, after interacting with the Smad4 and translocating to the nucleus, the Smad2/3 and Smad complex regulates the transcription of genes such as E-cadherin, c-Myc, integrins, and interleukin-6 (IL-6). Latency-associated peptide (LAP) pro-domain, is attached to TGF-β, which is released in an inert state [115]. For the TGF-β signalling to occur, MMPs and conformational modifications caused by contacts with integrins break LAP. TGF-β is essential for the healing of wounds. During the inflammatory phase, platelets and leukocytes release TGF-β, which functions as a chemokine by encouraging fibroblast motility and advancement to the wound area. Furthermore, TGF-β1 stimulates angiogenesis, keratinocyte migration, and granulation tissue progress, throughout the proliferative phase. TGF-β1 promotes the transition of fibroblasts into myofibroblasts over the remodeling phase, as evidenced by the elevation of α-smooth actin, which in turn promotes collagen synthesis and contraction of wounds. In case of diabetic foot ulcer, the expression of TGF-β1 is dysregulated which leads to delay in healing and closure of wounds. Hence, several methods and strategies are being proposed to increase the TGF-β1 expression and stimulate chronic wound healing [117].

### 4.3 CXCR4-induced Chemotaxis, Proliferation, and Cell Survival

#### 4.3.1 Cell Survival

Cell survival during wound healing in diabetes is a crucial component that is influenced by several diabetes-related variables [118]. Elevated blood glucose levels associated with diabetes can impede cellular viability by blocking the uptake of nutrients and oxygen, reducing the effectiveness of the immune system, and elevating cellular inflammation, all of which can impede the healing of wounds [119]. Diabetes can also result in neuropathy, which damages nerves and blood vessels and causes loss of sensation in certain places, especially the hands and feet. This can impair cell survival and wound healing [120]. One of the main biological modifications involved in tissue regeneration is apoptosis, which is controlled by CXCR4. In addition to cell death, physiological mechanisms including cell proliferation and chemotaxis are also involved in tissue regeneration [121]. Similar to cell division, growth, and proliferation, apoptosis is essential to the cell's life cycle. One of the most important aspects of wound healing is the removal of apoptotic cells brought on by the inflammatory milieu [121]. CXCR4 has a connection to cell death. Reduced expression of CXCR4 can cause cell death by blocking the PI3K/Akt/NF-κβ pathway. Additionally, pro-apoptotic or anti-apoptotic proteins can be affected by Akt activation through transcriptional or posttranscriptional mechanisms [40]. When SDF-1 binds to CXCR4 and CXCR7, it causes changes in cell adhesion molecules, the Rho-associated coiled-coil forming protein kinase (Rho-ROCK) pathway, anti-apoptotic signalling through B-cell lymphoma 2 (Bcl-2), and the epithelial-to-interstitial transition [40]. By blocking CXCR4, overexpression of miR-9-5p reduced the conversion of human umbilical cord endothelial cells caused by high sucrose, consequently decreasing the MAPK/ERK and PI3K/Akt/mTOR pathways [122]. Moreover, miR-146a can dose- and time-dependently downregulate CXCR4 expression. Phenotypic experiments have demonstrated that miR-146a mimics target CXCR4 to inhibit cell migration and proliferation and cause apoptosis [123]. The expression of CXCR4 is correlated with the transforming growth factor beta/interleukin 6 (TGF-β/IL-6) ratio. Furthermore, TGF-β and IL-6, two cytokines, are involved in senescence-promoting CXCR4 induction and lymphocyte differentiation [124].

#### 4.3.2 Cell Proliferation

Cell growth, which is controlled by CXCR4, is what makes tissue grow again. The proteins SDF-1 and CXCR4 seem to play a big role in helping diabetes wounds heal by increasing the number of hepatocytes. A new *in vitro* study says that SDF-1 can make cytokines work better on normal myeloid progenitor clonal growth [125]. Furthermore, SDF-1 may be important for epithelialization because it helps epidermal stem cells migrate and multiply. Several signal axis routes help cells divide and grow, and CXCR4 seems to be a part of a number of these [126]. Mitogens can also cause cells to divide, which is related to the MAPK pathway. When CXCR4 interacts with its ligand, the receptor may be taken inside the cell and MAPK may be turned on. This may help the cell divide. A different study says that extracellular signal-regulated kinase (ERK) is turned on to make cells multiply when SDF-1 is present [127]. Also, MAPK kinase-1/2 signals can directly activate ERK1/2 when SDF-1 is present; Gαi or new protein synthesis is not needed. By stopping CXCR4/ERK from activating, it can stop the growth and movement of cells caused by far-infrared radiation (FIR) [128]. FIR can increase the amounts of Nanog, NKX2-5, cluster of differentiation-117 (c-Kit), CXCR4, SOX-2, and other genes at the mRNA and protein levels [129]. This helps in cell migration and proliferation.

#### 4.3.3 Chemotaxis

An organism's reaction to chemicals in its surroundings is known as chemotaxis, which allows cells to move to areas where pertinent chemicals are present and perform related biological functions [130, 131]. Cell chemotaxis, facilitated by directed cues from chemokines and their receptors, enables cell migration, an essential component of tissue repair. During wound healing, immune response cells such as MSC mesenchymal stem cells, and lymphocytes serve as the main sites of cell migration. In the processes of tissue repair and regeneration, cell migration is a highly structured, multi-step process, and chemokines are primarily involved in lymphocyte trafficking [132, 133]. Chronic wounds require the migration of stem cells to the affected area to replenish lost or damaged cells [62]. One of the most important chemokines of stem cells is SDF-1, a ligand for CXCR4, which can be essential for mending damaged tissue and healing wounds [134-136]. The most studied chemokine receptor axis in MSC homing is CXCR4 and its ligand SDF-1. Increasing CXCR4 has been shown to enhance migration to SDF-1 through *in-vitro* chemotaxis testing [137].

## 5 REGULATION AND MODULATION OF CXCR4

### 5.1 CXCR4 Activation and Desensitization Mechanisms

#### 5.1.1 CXCR4 Activation

In response to CXCL12/SDF-1α, CXCR4 often activates G proteins that are members of the Gi subfamily. Gi protein activation suppresses AC, cAMP levels within the cell, and cAMP-dependent kinases such as PKA activity [43]. It also initiates the release of Ca^2+^ by the activation of PLC-β *via* the Gβγ subunits and the activation of PI3K, which in turn stimulates the ERK and Akt pathways [138]. These many signaling channels result in a variety of biological outcomes, such as cell migration, adhesion, proliferation, and survival [47]. CXCR4 binds to and activates the G protein subtypes Gi1, Gi2, Gi3, and Gio in response to CXCL12 stimulation *via* bioluminescence resonance energy transfer (BRET). It was found that CXCR4 couples with the Gi1 and Gi2 subtypes more effectively than with the Gi3 and Gio subtypes [139]. Gz has not been seen to activate. CXCR4 uses G13 and Gq proteins to signal in specific situations. On the one hand, turning on Rho through G13 and Rac limited the movement of Jurkat T cells that were activated by CXCL12/CXCR4 [140]. This axis was important for spreading basal-like breast cancer cells to other parts of the body. Furthermore, the Rho/G13 axis regulates CXCL12-induced CXCR4 trafficking into Rab11^+^ endosomes [141]. Conversely, CXCL12-triggered T cell hybridoma invasion and spread in mice required Gq proteins. CXCL12/CXCR4 triggered chemotaxis in granulocytes and dendritic cells, which also required their presence [142]. After CXCL12 activation, phosphorylation of CXCR4 attracts β-arrestin-1/2, which in turn causes the receptor to internalize, with β-arrestin-2 being the primary mechanism. In this way, lymphocytes from β-arrestin-2 knock-out mice had more G protein coupling of CXCR4 and less desensitization [143]. Both β-arrestins have been shown to greatly increase p42/44 MAPK activation through CXCR4 in response to CXCL12. This leads to more cell growth and survival, which is in line with their ability to boost signaling. By activating p38-MAPK, β-arrestin-2 also controls the chemotactic response of cells [144]. The C-terminus and ICL-3 are two different parts of CXCR4 that interact with each other. These two parts seem to be how β-arrestin affects the function of CXCR4 [145]. Additionally, CXCR4 can momentarily link to JAK-2/3, which activates and translocates several STAT proteins to the nucleus [146].

#### 5.1.2 Desensitization Mechanisms

Three methods exist to stop CXCR4 signalling: de-sensitization, internalization, and degradation, which serve as preventative measures against improperly prolonged signaling. Desensitization of CXCR4 and other GPCRs can happen quickly or over time. β-arrestin blocks G-protein engagement, causing short-term desensitization that lasts for minutes [43]. Prolonged desensitization takes place for hours to days and is caused by ongoing degradative processes, transcriptional downregulation, and internalization [147]. Once the ligand binds, it phosphorylates CXCR4, bringing in G-protein. Arrestin then intervenes to prevent G-protein from interacting with the receptor, a process known as desensitization. Ligand binding is used to control receptor internalization [148]. According to recent research, inverse agonists (TAK779) of CXCR4 induce receptor internalization at a greater rate than partial agonists (Met-CCL5). It suggests an active drive for the internalization process. Once internalized, CXCR4 signalling can proceed. For instance, by binding CXCR4, which is located in the endosome, CXCL12 promotes the survival of human neural progenitor cells [149]. Additionally, CXCR4 localizes to the endosome for many stages of regulated degradation. Cytoskeletal activity can be linked to endosomal degradation and CXCR4 internalization [150]. Actin chains, formed by Rho and Gα13, facilitate the movement of CXCR4 into the endosomal compartment. Endocytosis also requires adaptor proteins [151]. For example, CXCR4, β-arrestin, and the adaptor protein (AP2) work together to help CXCR4 endocytosis [152].

### 5.2 Influence of Factors TGF-β on CXCR4 Regulation

Fibroblasts are the primary cell type that drives both wound recovery and tumor stroma. Fibroblasts are essential for tissue reconstruction following injury and contribute to wound closure, tissue remodelling, granulation tissue deposition, and ECM protein synthesis [153]. In wound healing, fibroblasts are triggered and proliferate into myofibroblasts, which emphasizes the shift that these cells go through from a dormant, inactive state to an activated one following tissue damage [154]. The prefix “myo” highlights the presence of α-smooth muscle actin (α-SMA), which is connected to these cells in injuries [155]. Fibroblasts within quiescence are spindle-shaped cells observed in homeostatic tissue. α1β1 integrin and fibroblast-specific protein-1 (FSP-1) transcription are linked to quiescent fibroblasts in mice [156]. When an injury develops or neoplasia appears, fibroblasts are attracted and stimulated in the surroundings milieu.

The variables that have been linked to fibroblast activation in both wound recovery and tumor stroma include integrin expression, connective tissue growth factor, FGF2, platelet-derived growth factor (PDGF), insulin-like growth factor (IGF), TGF-β, and hepatocyte growth factor (HGF) signalling [157]. TGF-β1 reinforces wound recovery through the SMAD-4 system, comprising wound closure by fibroblast contraction, immune cell recruitment, angiogenesis, ECM protein deposition, and keratinocyte movement through integrins. TGF-β2 promotes ECM deposition, comparable to TGF-β1, but TGF-β3 modulates TGF-β1 and suppresses scar development and collagen-I accumulation [158]. TGF-β induces immune system cells to secrete CXCR4, resulting in greater migration to wounds. However, in diabetic-related wound repair, the utilization of CXCL12 limits the infiltration of endothelial progenitor cells [159]. In diabetic mice, CXCL12 therapeutics and oxygen administration significantly improved wound healing. The CXCL12 antagonist was injected into diabetic mice, and this impeded wound repair, specifically in the early phases [159]. In such wounds, there was decreased tissue granulation and angiogenesis, and higher concentrations of the pro-inflammatory cytokines (IL-6) and chemokines (CCL8). The lower expression of CXCL12 in diabetic-related wounds could contribute to the documented impaired wound healing [160].

Treatment with CXCR4 agonists enhanced angiogenesis and shortened the wound contraction phase in diabetic mice by 36%. In diabetic fibroblasts, the agonist also decreased the overexpression of miRNA-29a and miRNA-15b, which are negative effects of collagen synthesis and angiogenesis, respectively [161]. To sum up, TGF-β increases the expression of CXCR4, which encourages the recruitment of immune cells to wounds. Nevertheless, wound healing and the migration of progenitor cells are compromised by decreased CXCL12 expression in diabetic wounds. CXCR4 agonists have the potential to improve the healing of diabetic wounds by increasing the production of collagen and angiogenesis [161].

## 6 THERAPEUTIC STRATEGIES TARGETING CXCR4 IN DIABETIC WOUND HEALING

The literature review in Table **1** shows the importance of CXCR4 pathways in diabetic wound healing and the potential benefits of targeting this pathway. Based on the advantages we proposed the novel pathway CXCR4 role in wound healing and CXCR4 role in involvement of exosome-mediated wound healing. The clinical data is still lagging behind the need for additional explication of specific pathways, and the fact that some research has focused on generalized wound healing rather than wounds caused by diabetes are among the limitations.

### 6.1 Overview of CXCR4 Targeted Therapies

The skin wound-healing system is dynamic and multifaceted, however, it is also highly structured, with structural events that overlap across phases that involve various cells and factors. The CXCR4 is one of the many chemokine receptors which is involved in inflammation and wound healing. This CXCR4 can be enfolded by SDF-1, CD74, and CD4. SDF-1 could potentially be the only intrinsic ligand of CXCR4 [21]. CXCR4 plays a crucial involvement in both biological processes including neurogenesis, cardiogenesis, vascular formation, and germ cell development along with pathological processes such as vascular formation, and muscle regeneration [20]. CXCR4 is upregulated in the body through injury, stress hypoxia, and vascular tissue injury [21]. Also when tissues such as the kidney, liver, brain, and heart are impaired, the level of SDF-1 can expressively upregulate. CXCR4-positive stem cell/precursor cells are induced to express early tissue markers in the bone marrow and partake in wound repair and regeneration during injury [166, 167]. The major physiological processes allied with wound healing can be initiated by the SDF-1/CXCR4 axis such as chemotaxis of inflammatory cells to impaired tissues, collagen deposition for tissue remodeling, and cell proliferation for wound repair [65, 168]. Several signaling pathways are activated by the SDF-1/CXCR4 signaling cascade, involving nuclear factor-activated light chain enhancer B cells (NF-κB), which regulates intracellular transcription, Ca^2+^ efflux, and cell survival, and PI3K/Akt, mTOR, and JAK/STAT cascade [169]. The SDF-1/CXCR4 signaling axis therefore regulates the fundamental aspects of cellular differentiation, cellular survivability, chemotaxis, and apoptosis, which can also advance angiogenesis in certain disorders. Thus, it is crucial for the healing of wounds [170].

### 6.2 Promising Results of CXCR4-based Molecules

#### 6.2.1 AMD3100

Inflammation is a fundamental aspect of the healing wound mechanism and mediates many of the effects brought by chemokines such as SDF-1/CXCR4 and controlling these components will control both cell migration and inflammation [171, 172]. Therefore, SDF-1 and CXCR4 interactions will stimulate in the healing of cutaneous wounds. So, a therapeutic approach that hinders the interaction between SDF-1 and CXCR4 is therapeutic interventions that show potential for a range of clinical uses [173]. AMD3100 is the first CXCR4 antagonist to be explored in clinical studies. By accelerating the infiltration of endothelial progenitor cells (EPCs) from the bone marrow to the rejuvenated vasculature following myocardial infarction, the CXCR4 antagonist AMD3100 improves cardiac functionality [174]. Following a week of therapy, AMD3100 was related to increased numbers of circulating macrophages and EPCs as well as distinct increased mRNA levels of SDF-1 and PDGF-B in the wound site [87]. Additionally, this AMD3100 induced the migration and proliferation of diabetic mice with initial dermal fibroblasts and 3T3 fibroblasts, which express less CXCR4 and it also increased the phagocytosis and proliferation of macrophages as shown in Fig. (**5**) [175]. With just a single topical treatment in diabetic mice with AMD3100, accelerated wound healing by advancing cytokine production, mobilizing bone marrow EPCs, and activating fibroblasts and monocytes or macrophages, causing in increased vasculogenesis and angiogenesis; but not all AMD3100 action is through the antagonizing of CXCR4 [87].

#### 6.2.2 UCUF-728

In conditions like cancer, inflammation, HIV, and diabetes, the G-protein coupled receptor (GPCR) CXCR4 and its intrinsic ligand CXCL12 are known to be dysregulated [176]. In case of hypoxia, vascular tissue damage, stress, and injury, the CXCR4 and CXCL12 are upregulated [21]. A systematic and synchronized process is involved in wound healing like epithelialization, granulation tissue formation, angiogenesis, and wound narrowing. It needs the cooperation of several cell types, growth factors, proteins found in the ECM, and enzymes [177]. To initiate chemotaxis to injured tissues, cell proliferation for repair, and the accumulation of collagen for tissue remodeling, the CXCL12/CXCR4 axis is necessary for wound healing [21]. Increased narrower epithelial gap, granulation tissue, and a larger wound are the outcomes of overexpressing CXCL12. In contrast, the expression of a mutant form of CXCL12 inhibits the activation of CXCR4 by endogenous CXCL12 and causes a significant increase in inflammation, a decrease in angiogenesis, and a slowdown in the healing of wounds in diabetic mice [178]. Impaired wound healing is correlated with reduced expression of CXCL12 in diabetics. Small molecule medicinal discovery and development may be facilitated by activating CXCR4 receptor signaling with agonists or positive allosteric modulators (PAMs). Recent research using high-resolution screening identified UCUF-728 as a partial agonist of CXCR4, and it speeds up the healing process in diabetic mice when administered at 100 μM [161]. In further studies, the CXCR4 agonist UCUF-728 in an *in-vitro* test show positive action on diabetic fibroblasts. In diabetic fibroblasts, UCUF-728 inhibited the overexpression of miRNA29a and miRNA-15b, which are negative regulators of angiogenesis and type I collagen synthesis, respectively (Fig. **5**). While an *in-vivo* study, the administration of UCUF-728 resulted in a reduction in wound repair time by 36% and an enhancement in angiogenesis activity in diabetic mice which exhibits the clinical action of small molecule CXCR4 agonists for pathologic wound healing in diabetes [162].

#### 6.2.3 UCUF-965

The development of a synthetic structure with increased agonist efficacy was a thiadiazine variant, UCUF-965. This pharmacologic molecule promotes β-arrestin trafficking in a CXCR4 receptor overexpressing cell line. UCUF-965 enhanced the total CXCL12 maximal response in the cAMP signaling cascade and stimulated CXCL12-induced migration in lymphoblast cells in a multi-level screen. It also regulates specific microRNAs that participate in the multi-factorial and multi-level wound repair process in fibroblasts from the mouse [179]. UCUF-965 acts as a new positive allosteric modulator (PAM) agonist of the CXCR4 receptor. Subsequently, in diabetic C57BL6 mouse models, UCUF-965 strongly enhanced angiogenesis markers and hastened wound repair by 36%. Interestingly, *versus* untreated control, it shortened wound repair time to 6.4 days without impacting the number of pro-angiogenic tissues by 59.3% and 56.7%, respectively (Fig. **5**) [179].

### 6.3 Other Emerging Therapies in the Field of Diabetic Wound Healing

#### 6.3.1 Complement Growth Factors

Wound healing is a complicated process that involves several growth factors, some of which have various impacts on the distinct cell types. PDGF is a major agent that is active throughout healing process. Becaplermin (0.01% Regranex gel), a homodimeric protein that is generated using recombinant DNA technology by inserting the B chain PDGF gene into the yeast *Saccharomyces cerevisiae* [180]. The enhancement of chemotactic selection and the proliferation of cells that are essential in wound healing are the two biological activities that becaplermin shares with native PDGF-BB. Becaplermin has passed extensive tests on both humans and animals, showing that when taken in conjunction with conventional wound healing procedures, it is a highly effective supplementary therapy for the healing of ulcerations in diabetic feet. These procedures specifically involve monitoring infection, removing as much weight as possible from the tormented region, and providing a moist environment that is devoid of debris and necrotic tissue. Becaplermin is currently the only growth factor that is approved for use in wound healing. It is safe and simple to use, requiring only one application daily [180].

#### 6.3.2 Inflammatory Regulators

Diabetes is an inflammatory illness that typically results in chronic sores for which there are presently no effective treatments. In diabetes, there is a low nitric oxide (NO) concentration in wounds, which impairs wound healing. According to a new study, diabetic wounds can be physically targeted utilizing cold atmospheric plasma (CAP) to control the inflammatory state of the wounds. Exogenous, gaseous NO may be readily applied to the wounds using CAP therapy, and the quantity can be managed by altering the CAP concentration [181].

Clinical research, animal studies, and cell investigations have all shown how well gaseous NO promotes diabetic wound healing and there have been no reports of pain or adverse outcomes from clinical studies. The utilization of NO concentrations lower than 200 ppm for wound healing is a common characteristic, which is associated with the dual regulation of NO on inflamed and the regulation of macrophage polarization [182]. A thorough examination of inflammatory variables is being carried out, as well as physical research into the properties of the helium plasma jet. The analysis is also done on the CAP jet's potential for clinical use on human bodies as well as its biological and physical safety. Instead of substantially interfering with a particular target, the results indicate that CAP treatment can promote the body's natural inflammatory regulatory mechanism to attain a normal condition. This entails the stimulation of anti-inflammatory factors in the resolution sub-phase that follows the suppression of pro-inflammatory factors during the onset sub-phase [183].

#### 6.3.3 Hyperbaric Oxygen Therapy

Chronic non-healing ulcers can be resistant to conventional therapy, and any individual with DFUs cannot be repaired with surgical debridement, which necessitates alternate options for therapy. One adjunct therapy that has been utilized for decades to treat complicated DFUs is hyperbaric oxygen therapy (HBOT) [184]. Oxygen is essential for the healing of chronic wounds since the hypoxia in infected tissues prevents ulcer repair. For optimal therapeutic results, the patient receiving HBOT is housed in a chamber with 100% breathing oxygen at an atmospheric pressure higher than sea level, typically 1.4 atmospheres absolute. Numerous positive physiological results which include reduced edema, increased angiogenesis, increased leukocyte activity, and enhanced collagen deposition, were seen in patients who are being treated with HBOT. So, by raising the oxygen content in tissues, HBOT helps to speed up the healing of ulcers and further reduces the risk of amputations [185].

#### 6.3.4 Skin Replacement Therapy

Skin replacements are classified into subgroups according to their composition, derivative source, and distinguishing ingredients. These consist of implantable scaffolds that include an epidermal or dermal component. If the biomaterials utilized are entirely synthetic, sourced from a biological source, or a combination of the two, further differentiation might be seen. Interestingly, though, a lot of effective skin replacements are made of a range of separately derived ingredients, which obscures the complete method of contribution of each component [186]. This is particularly relevant considering the complexity of surroundings with chronic wounds and the individual's clinical diversity on both a local and systemic scale. Dermal element scaffolds, in certain entirely acellular dermal matrices, are used in diabetic foot ulcer treatment because of their induced therapeutic mechanistic effects related to ECM, prevalence, history of positive clinical results, safety regarding tissue rejection, and profusion in recent clinical trials [187].

#### 6.3.5 Stem Cell Therapy

One potentially effective therapeutic option for diabetic wound regeneration is stem cell therapy. The different cytokines and growth factors produced by stem cells have been shown to play a significant role in immunomodulatory activities and regeneration, which are typically physiologically weak in chronic wounds, making stem cells an attractive and suitable therapy choice [188]. There are various types of stems cells in diabetic wound healing namely embryonic stem cells (ESCs), induced pluripotent stem cells (iPSCs), mesenchymal stem cells (MSCs), and adipose-derived stem cells (ADSCs).

ESCs, also known as PSCs, can develop into several cell types, such as neuronal, blood, chondrocyte, adipocyte, skin cells, and muscle. This exceptional regeneration capability has attracted interest for use in cutaneous regeneration and wound closure. Since the cells are separated from the living embryo, the only restrictions are those related to religion, ethics, and the law [189].

The most advanced type of PSC is called iPSC. Human cells were used to create iPSCs, and these cells had many characteristics with ESCs, including pluripotency, growth, and shape. By eliminating the major drawbacks associated with using ESCs, such as immune rejection and ethical problems, this innovative approach allows for the use of “autologous” PSC groups in regenerative therapies. Several preclinical investigations have found that iPSCs-induced mesenchymal stem cells (iMSCs) help to regenerate and repair blood vessels, liver, periodontal tissue, heart, chondrocytes, cutaneous wounds, and skeletal muscle [190].

Among the MSCs, those derived from cutaneous and especially adipose tissue have been highlighted, as they are stable MSC sources easy to obtain. Adult MSC is the classic adult stem cells that self-regenerate and specialize. As they have been functionally implicated in organ homeostasis and wound reparative, there are indications for their benefit against a variety of diseases [191]. Some research was carried out regarding bone marrow stem cells and their ability to accelerate diabetic wound healing in the setting of chronic non-healing pressure ulcers in diabetes control, through suppression of anti-inflammatory conditions, improvement tissue angiogenesis and granulation formation while other experiments implicated MSC in promoting cell proliferation for re-epithelialization. In addition, it was established that EGF-stimulated MSCs can restore the impaired blood flow in diabetic mice presumably *via* a VEGF receptor-dependent mechanism of inducing neovascularization [192].

The ASCs which are very numerous and accessible among MSCs, are widely known as a different alternative for employment in the repair of diabetic wounds. ASCs may develop into many cell lineages, including adipogenesis, myogenesis, osteogenesis, and chondrogenesis but also secrete a variety of growth hormones, including as (VEGF, FGF, and HGF), to encourage cell development and quicken wound healing [193].

## 7 LIMITATIONS OF CXCR4 THERAPY FOR WOUND HEALING IN DIABETIC PATIENTS

Dysregulation of the CXCR4/CXCL12 axis may compromise wound healing in diabetes patients. Diabetes significantly lowers CXCL12 expression, leading to heightened inflammation and postponed wound closure. Because of the hematopoietic cells required for tissue repair caused by this low expression, the healing process takes longer and the likelihood of chronic wounds increases [162]. CXCR4 activation may unintentionally encourage excessive inflammation. Even though chemotaxis and the growth of cells that help wounds heal depend on CXCR4, too much of this receptor can cause an overactive inflammatory response. This may lead to the extended persistence and activation of macrophages and neutrophils, which could exacerbate tissue injury instead of aiding in its healing. Furthermore, a complicated interaction between the feedback loop and other signaling pathways, notably PI3K/Akt and mTOR, may result in cancer and other unforeseen effects [21]. There is concern that people with chronic diabetic wounds may become more susceptible to cancer because the pathways that CXCR4 activates are similar to those involved in cancer cell growth. The possibility of reduced cellular recruitment and migration is one major worry. CXCR4 facilitates the recruitment of hematopoietic progenitor cells to injury sites, which is essential for effective wound healing. Because CXCR4 inhibition can mess up this chemotactic response, cells may not move around as much, which is needed for tissue repair. This can lead to delays in wound closure and a higher chance of chronic wounds, especially in people with diabetes who already have low CXCL12 levels [164]. Furthermore, CXCR4 inhibition can result in inadequate inflammatory responses that are required for wound healing, even if it can potentially lessen excessive inflammation in some situations. The right amount of inflammation is essential; too little can make it more difficult for immune cells to recruit and aid in the removal of debris and infection prevention, while too much inflammation can lead to tissue damage. Inhibiting CXCR4 may tip this equilibrium, leading to an insufficient immune response and heightened vulnerability to infections. Furthermore, the CXCR4 signaling pathway not only facilitates wound healing but also plays a role in angiogenesis and tissue regeneration, among other physiological activities [194]. Blocking this pathway can lead to reduced blood vessel formation and worse nutrient delivery to the wound site. This may make healing more challenging, particularly in regions where regeneration depends on a strong circulatory supply. One runs the risk of encouraging carcinogenesis. Inhibiting CXCR4 may unintentionally alter normal tissue homeostasis and increase the risk of cancer in those who are vulnerable, given its link to the growth and spread of cancer cells [164]. Due to its dual function, CXCR4 complicates treatment plans, as blocking this route may have unintentional carcinogenic effects.

## CONCLUSION

Diabetes presents a significant impediment to wound healing due to intricate relationships between reduced immune function, neuropathy, and a compromised circulatory system. Many recent studies and clinical experiences have highlighted several critical aspects and identified their role in managing diabetic wounds most effectively. A reduced vasculogenesis in diabetic wounds may lead to insufficient oxygen and nutrient supply which are both important for healing. Additionally, a protracted inflammatory phase in diabetic wounds prevents healing and slows progress toward the proliferation phase. The wounds are more susceptible to infections at this stage since the immune system is weak and the hyperglycaemic environment promotes pathogen proliferation. Diabetes patients who lose their sensation often have tiny cuts that go unnoticed and unreported, and then develop into ulcers. Chronic infections contribute to prolonged inflammatory responses and necrosis, further complicating wound healing and often necessitating major medical interventions like amputation of the affected foot or part of the body. CXCR4 is a receptor that regulates numerous factors, like inflammation, cellular signaling pathways, microbial invasion, and necrosis which have a significant impact on the complications of diabetic wound healing due to the dysregulated of CXCR4 that can cause impairment in the wound healing process. Hence, it is essential to comprehend these elements and how they interact to formulate effective treatment procedures. Several aspects of wound healing are regulated by the CXCR4 and its ligand, SDF-1. Effective wound healing depends on CXCR4 signaling like cell proliferation, migration, and persistence. Two reported intracellular pathways can trigger CXCR4 signaling, specifically PLC/PKC-Ca^2+^ and MAPKs. These pathways facilitated the wound healing process *via* the proliferation, chemotaxis, and endurance of cells. Many variables, like TGF-β and HER2 (human epidermal growth factor receptor-2) firmly control and influence the expression of CXCR4, and the dysregulation of CXCR4 by therapeutic intervention targeting is essential in diabetic patients. So comprehending the processes and pathways underlying CXCR4 dysregulation can assist in the development of modified targeted drug delivery and enhance the wound healing outcomes in diabetic individuals. The process of SDF-1 binding to CXCR4 initiates downstream signaling pathways, which are fundamental to regulating the cellular processes that are required for wound healing. To establish appropriate CXCR4 activity and facilitate effective wound healing, approaches may incorporate the use of CXCR4 agonists or antagonists, gene therapy, or other molecular techniques. A thorough understanding of the molecular mechanisms bringing about diabetic wound healing is necessary, with an emphasis on the roles of microbial invasion, CXCR4 signaling, and inflammation. In the future, the formation of novel medicines that will alter or block the CXCR4 signaling pathway manages the inflammatory response by enhancing M2 macrophages and dysregulating overexpressed M1 polarization. By using the biomaterials increases the wound healing activity, which directly impacts macrophage polarization or the supply of CXCR4 ligands to the wound area in the future. These biomaterials were engineered to release the therapeutic drug under controlled conditions in local wound areas. These therapies may be associated with healing responses by re-establishing the balance of signaling pathways altered in diabetic wounds. Treatment plans could be tailored by combining knowledge from individual patient responses with regional patterns of inflammation. It will be essential to understand how various patients react to treatments that target the CXCR4 pathway to create efficient interventions. Thus, greater research on CXCR4 could be a crucial step toward facilitating tissue regeneration. Optimizing wound recovery and tissue regeneration may be achievable through controlling the SDF-1/CXCR4 signal axis. Hence, by focusing on these specific areas of research, distinctly through the alterations of CXCR4 action, novel treatment approaches can be developed to significantly enhance the wound healing process in a diabetic patient and future research should continue to investigate these interactions and molecular pathways.

## Figures and Tables

**Fig. (1):**
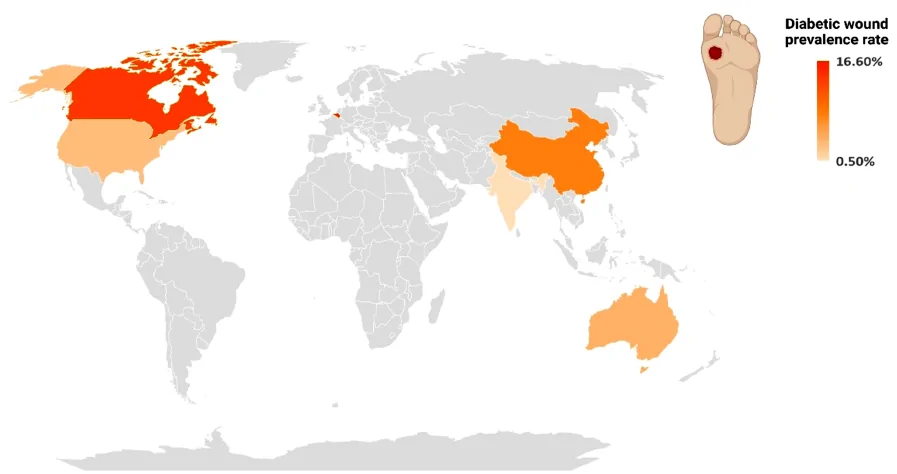
The prevalence of diabetic wounds varies in different regions as evidenced by the provided data.

**Fig. (2):**
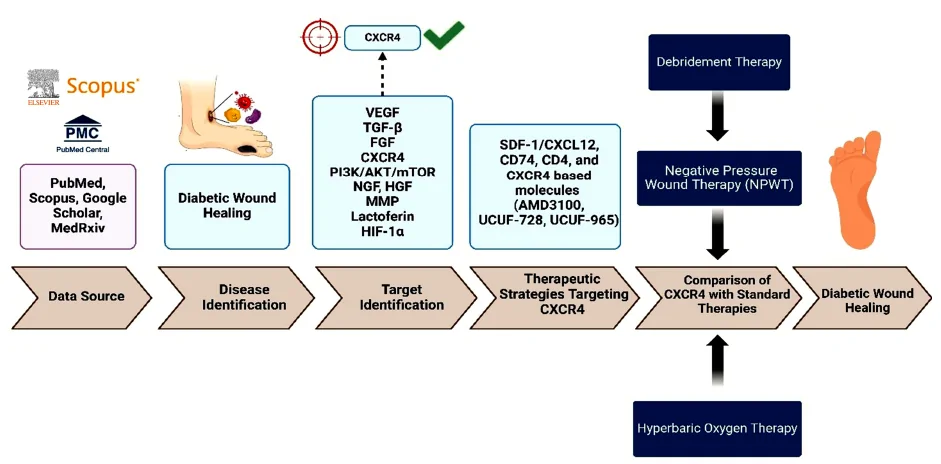
Algorithm or flow chart of the CXCR4-mediated diabetic wound healing.

**Fig. (3):**
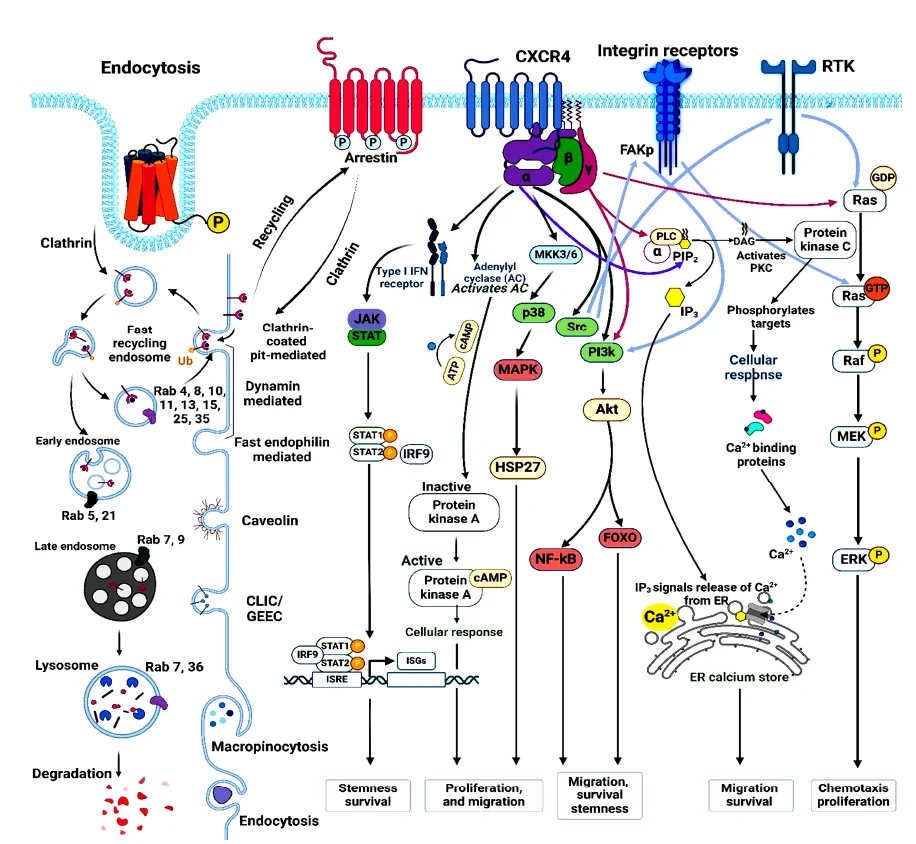
The complex series of CXCR4-mediated processes with a special emphasis on clathrin-mediated endocytosis. CXCR4 triggers a cascade of events upon activation by its ligand, starting with internalization through pits covered in clathrin. The internalized CXCR4 receptors then go through a quick recycling process *via* the fast-recycling endosome pathway. Arrestins are involved in the control of CXCR4 trafficking and signaling. The JAK/STAT pathway is then activated, and STAT proteins are important in signaling that occurs later on. Early endosomes are facilitated by dynamin, and it is inside them that CXCR4 is sorted for subsequent trafficking, which is controlled by distinct Rab proteins within distinct endosomal compartments. Alternative endocytic routes are also depicted, including caveolin-mediated and CLIC/GEEC pathways. It also emphasizes integration with integrin receptors. Activation of CXCR4 sets off downstream signalling cascades that involve transcription factors such as NF-κB and FOXO, adenylyl cyclase, MAPK, and PI3K/Akt. Numerous cellular responses, such as chemotaxis, proliferation, survival, and preservation of stemness and migratory capabilities, are the result of these signalling processes. A different pathway for CXCR4 internalization is provided by micropinocytosis, which results in cellular function modification and destruction. **Abbreviations: **PPP- Phosphoprotein phosphatase; JAK- Janus kinase; STAT- Signal transducer and activator of transcription; CLIC/GEEC- Clathrin-independent carriers/GPI-AP enriched early endosomal compartment; CXCR4- C-X-C chemokine receptor type-4; AC- Adenylyl cyclase; IRF9- Interferon regulatory factor-9; FAKP- Focal adhesion kinase-related non-kinase; RTK- Receptor tyrosine kinase; GD- Guanosine diphosphate; PLC- Phospholipase C; MKK3/6- Mitogen-activated protein kinase 3/6; PIP2- Phosphatidylinositol 4,5-bisphosphate; DAG- Diacylglycerol; PKC- Protein kinase C; PI3K- Phosphoinositide-3-kinase; HSP27- Heat shock protein-27; PKA - Protein kinase A; NF-κB- Nuclear factor kappa-light-chain-enhancer of activated B cells; FOXO- Forkhead box O; ISGS- Interferon-stimulated genes; GTP- Guanosine triphosphate; IP3- Inositol trisphosphate; Raf P- Phosphorylated Raf; MEKP- MAPK/ERK kinase phosphatase; Ca^2+^ BP- Ca^2+^ binding proteins; and ERK- Extracellular signal-regulated kinase.

**Fig. (4):**
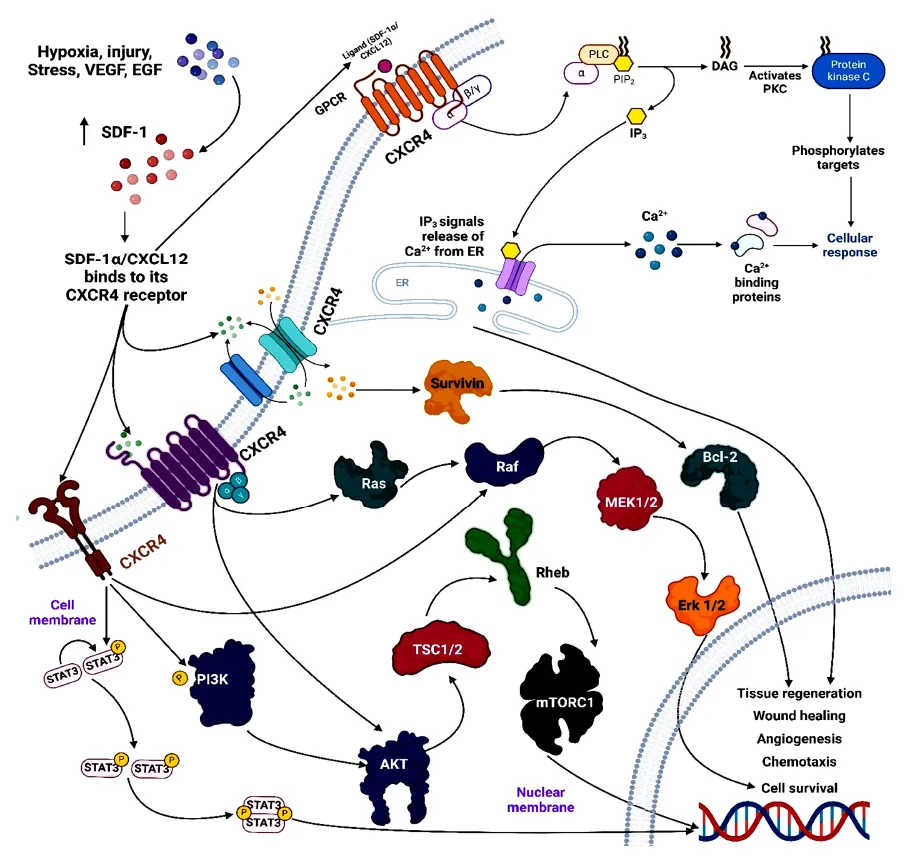
Schematic representation of SDF-1/CXCR4 is involved in intracellular signal transduction pathways that are linked to wound healing. The expression of CXCR4 on the cell membrane is influenced by a variety of *in-vivo* and *in-vitro* stimuli, which also control the CXCR4 signalling pathway and take part in multiple wound repair processes, such as tissue regeneration, angiogenesis, and cell survival. **Abbreviations: ** VEGF- Vascular Endothelial Growth Factor; EGF- Epidermal Growth Factor; SDF-1- Stromal Cell-Derived Factor 1; CXCL12- C-X-C Motif Chemokine Ligand 12; CXCR4- C-X-C Chemokine Receptor Type 4; GPCR- G-Protein Coupled Receptor; PLC- Phospholipase C; PIP2- Phosphatidylinositol 4,5-bisphosphate; DAG- Diacylglycerol; PKC- Protein Kinase C; IP3- Inositol trisphosphate; ER- Endoplasmic Reticulum; Bcl-2- B-cell lymphoma 2; MEK1/2- Mitogen-Activated Protein Kinase Kinase; Rheb- Ras homolog enriched in brain; Erk ½- Extracellular Signal-Regulated Kinase; TSC1/2- Tuberous Sclerosis Complex; PI3K- Phosphoinositide 3-Kinase; AKT- Protein Kinase B; and mTORC1- Mammalian Target of Rapamycin Complex-1.

**Fig. (5):**
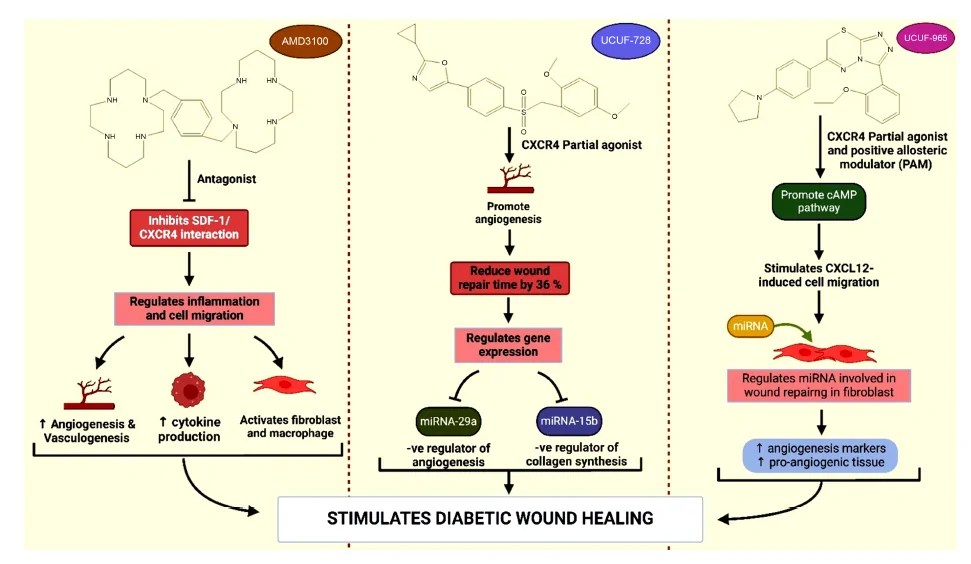
The basic processes by which compounds targeting the CXCR4 receptor function in diabetic wound healing.

**Table 1:** Literature review of CXCR4 mediated wound healing in diabetic patients.

**Work Carried Out**	**Advantages**	**Disadvantages**	**References**
Researchers developed UCUF-728, a CXCR4 agonist, which reduced overexpression of miRNA-15b and miRNA-29a in diabetic fibroblasts and wound healing time in living things, suggesting a potential use for future diabetes-related wound treatments.	The clinical potential of targeting the CXCR4 receptor to enhance wound healing in diabetes patients is highlighted by this study. These results highlight a significant gap in existing therapeutics by suggesting that CXCR4 agonists, such as UCUF-728, may present a promising new strategy for treating chronic wounds in diabetes.	The study focuses on SDF-1 role in wound healing, but may overlook other factors and lack clinical trials, limiting its applicability in real-world settings and potentially not universally applicable to all diabetic patients.	[161]
Identified a triazolothiadiazine derivative (UCUF-965) that acts as a potent partial agonist and a positive allosteric modulator of CXCR4, enhancing angiogenesis and reducing wound healing time by 36% in diabetic mice at 10 μM.	Identified a novel chemical scaffold with increased potency compared to previous compounds. Demonstrated efficacy in accelerating wound healing in diabetic mice.	Only tested in mouse models, not yet in humans. Specific mechanisms of action not fully elucidated.	[162]
Reviewed the pleiotropic roles of CXCR4 in wound repair and regeneration, including activating chemotaxis, cell proliferation, and collagen deposition.	Provides a comprehensive overview of the various roles of CXCR4 in wound healing. Highlights the potential of targeting CXCR4 for therapeutic interventions.	It is mainly a review article, not presenting new experimental data. Focuses on general wound healing, not specifically diabetic wounds.	[21]
Discussed the effects of various exosomes on diabetic wound healing *via* different signaling pathways, including the CXCR4 pathway.	Provides a mini-review on the role of exosomes in promoting diabetic wound healing covers multiple signaling pathways involved in the process.	It is mainly a review article, not presenting new experimental data. Focuses on exosomes, not directly on CXCR4 signaling.	[163]
Targeting CXCR4 improved wound healing in mice. Reduced inflammation and fibrosis.	It increased CXCR4 expression in diabetic wounds. Activation of downstream signaling pathways (ERK, Akt). Impaired wound healing in mice.	Limited sample size. *In-vitro*, findings may not fully translate to *in- vivo* conditions.	[164]
This study investigates the therapeutic potential of CXCR4/IL-10 dual angiogenic/anti-inflammatory gene knock-in amniotic mesenchymal stem cells (AMSCs) for enhancing wound healing. Using transcription-activator-like effector nuclease (TALEN) technology, CXCR4 and IL-10 genes were inserted into the AMSCs genome. *In-vitro* assessments demonstrated that CXCR4/IL-10-expressing AMM/CI exhibited robust tube formation and anti-inflammatory properties. *In-vivo* experiments in a diabetic wound-healing model showed that AMM/CI transplantation significantly accelerated wound healing, achieving high rates of engraftment and re-epithelialization.	The advantages of this approach include enhanced angiogenesis and reduced pro-inflammatory gene expression in wound tissue, indicating direct therapeutic effects.	The study also presents disadvantages, such as the complexity of gene editing techniques and the need for further research to determine the long-term safety and efficacy of AMSCs/CI in clinical applications.	[165]

## LIST OF ABBREVIATIONS

AMPs: Anti-microbial Peptides
ADP: Adenosine Diphosphate
ATP: Adenosine Triphosphate
ADSCs: Adipose-derived Stem Cells
AP2: Adaptor Protein-2
BHLH: Basic Helix-loop-helix
BMSCs: Bone Marrow-derived Mesenchymal Stem Cells
Bcl-2: B-cell Lymphoma 2
BRET: Bioluminescence Resonance Energy Transfer
cAMP: Cyclic Adenosine Monophosphate
CD4: Cluster of Differentiation 4
CXCR4: C-X-C Chemokine Receptor 4
cPLA2: Cytosolic Phospholipase A2
CAP: Cold Atmospheric Plasma
DAG: Diacylglycerol
DDR: DNA Damage Response
ECM: Extracellular Matrix
ERKs: Extracellular Signal-regulated Kinases
ESCs: Embryonic Stem Cells
EPCs: Endothelial Progenitor Cells
ER: Endoplasmic Reticulum
FOX: Forkhead Box
FGF: Fibroblast Growth Factor
FIR: Far-infrared Radiation
FSP-1: Fibroblast-specific Protein-1
GRK2: G-protein-coupled Receptor Kinase 2
GSK-3: Glycogen Synthase Kinase-3
GF: Germ-free
HBOT: Hyperbaric Oxygen Therapy
HEK293: Human Embryonic Kidney 293
HER2: Human Epidermal Growth Factor Receptor-2
HGF: Hepatocyte Growth Factor
IGF: Insulin-like Growth Factor
iPSCs: Induced Pluripotent Stem Cells
IL-1β: Interleukin-1β
INF-γ: Interferon-γ
IP3: Inositol 1,4,5-trisphosphate
IL-17: Interleukin-17
IVD: Intervertebral Disc
JAK/STAT: Janus kinase/Signal Transducer and Activator of Transcription
JNKs: jun N-terminal Kinases
LAP: Latency-associated Peptide
MSCs: Mesenchymal Stem Cells
mTOR: Mammalian Target of Rapamycin
MAPK: Mitogen-activated Protein Kinase
MMP-2: Matrix Metalloproteinase-2
miRNA-21: Micro RNA-21
MKK6: MAPK Kinases 6
NOD2: Nucleotide-binding Oligomerization Domain-2
NO: Nitric Oxide
NF-κB: Nuclear Factor Kappa B
NRF-1: Nuclear Respiratory Factor-1
PAMs: Positive Allosteric Modulators
PDGF: Platelet-derived Growth Factor
POU: Pit-Oct-Unc
PI3K: Phosphoinositide-3 Kinase
PKB: Protein Kinase-B
PLC: Phospholipase-C
PLD: Phospholipase-D
PIP2: Phosphatidylinositol 4,5-bisphosphate
PKC: Protein Kinase-C
PPF: Particular Pathogen-free
PTX: Pertussis Toxin
pRb: Retinoblastoma Protein
PGC-1α: Peroxisome Proliferator-activated Receptor Gamma Co-activator-1α
Rho-ROCK: Rho-associated Coiled-coil Forming Protein Kinase
ROS: Reactive Oxygen Species
SCID: Severe Combined Immunodeficiency
SOX17: Sex-determining Region Y-box 17
Smad-2: Suppressor of Mothers for Decapentaplegic
SDF1: Stromal Cell Derived-Factor-1
TNF-α: Tumor Necrosis Factor Alpha
TGF-β: Transforming Growth Factor-β
VEGF: Vascular Endothelial Growth Factor
YY1: Yin Yang-1
